# Maternal B12 deficiency during pregnancy dysregulates fatty acid metabolism and induces inflammation in human adipose tissue

**DOI:** 10.1186/s12916-025-04056-4

**Published:** 2025-04-23

**Authors:** Jinous Samavat, Joseph Boachie, Philip G. McTernan, Mark Christian, Ponnusamy Saravanan, Antonysunil Adaikalakoteswari

**Affiliations:** 1 Division of Health Sciences, Warwick Medical School, University of Warwick, Coventry, CV2 2DX UK; 2 Department of Medical Laboratory Technology, School of Allied Health Sciences, University of Cape Coast, Cape Coast, Ghana; 3 Department of Biosciences, School of Science and Technology, Nottingham Trent University, Nottingham, NG11 8 NS UK; 4 De Montfort University, The Newarke, Leicester, LE1 9BH UK; 5 Department of Diabetes, Endocrinology and Metabolism, George Eliot Hospital, Nuneaton, Warwickshire CV10 7DJ UK; 6 Centre for Global Health, Warwick Medical School, University of Warwick, Coventry, UK

**Keywords:** Vitamin B12, Adipose tissue, Low-grade inflammation, Lipid metabolism, Obesity, Pregnancy

## Abstract

**Background:**

Adipose tissue (AT) responds to excess calorie intake; however, the deficit in micronutrients accompanied by the modern lifestyle is often overlooked. Micronutrient deficiency in pregnancy, particularly vitamin B12 (B12), is commonly associated with higher adiposity, dyslipidemia, and type 2 diabetes (T2D). Studies have demonstrated that dyslipidemia can trigger pro-inflammatory status. However, the release of the pro-inflammatory factors in a tissue-specific micronutrient deficient environment is unexplored. Therefore, we investigated the role of B12 deficiency on lipid metabolism and inflammatory mediators in both in vitro and ex vivo models including human pre-adipocytes, primary adipocytes, mature human white AT (WAT), and its association with metabolic risk.

**Methods:**

Paired abdominal subcutaneous and omental WAT (ScWAT and OmWAT) were chosen based on serum B12 (< 150 pM) from 115 Caucasian pregnant women. Human primary Sc adipocytes from women with different BMI (lean, overweight, obese, morbidly obese) and pre-adipocyte cell line (Chub-S7) were differentiated in various concentrations of B12. Serum B12, folate, lipids, cytokines, biochemical parameters, gene expression, intracellular triglyceride (TG), and mitochondrial function were assessed.

**Results:**

In pregnant women with low B12 levels, BMI and serum TG were significantly higher, and high-density lipoprotein (HDL) was lower (*p* < 0.05). B12 deficiency in both depots of AT correlated with higher expression of genes in fatty acid (FA) synthesis, elongation, desaturation, TG synthesis, and reduced fatty acid oxidation (FAO) (*p* < 0.05). In vitro adipocytes with low B12 demonstrated that TG synthesis utilizing radiolabeled FA was higher and mitochondrial function was impaired. We also found that the expression of pro-inflammatory cytokines in AT was increased, and circulatory cytokines inversely associated with serum B12 (*p* < 0.05).

**Conclusions:**

Our novel data highlights that B12 deficiency dysregulates lipids and induces inflammation in AT and circulation, which could contribute to adipocyte dysfunction exacerbating cardiometabolic risk during pregnancy.

**Graphical Abstract:**

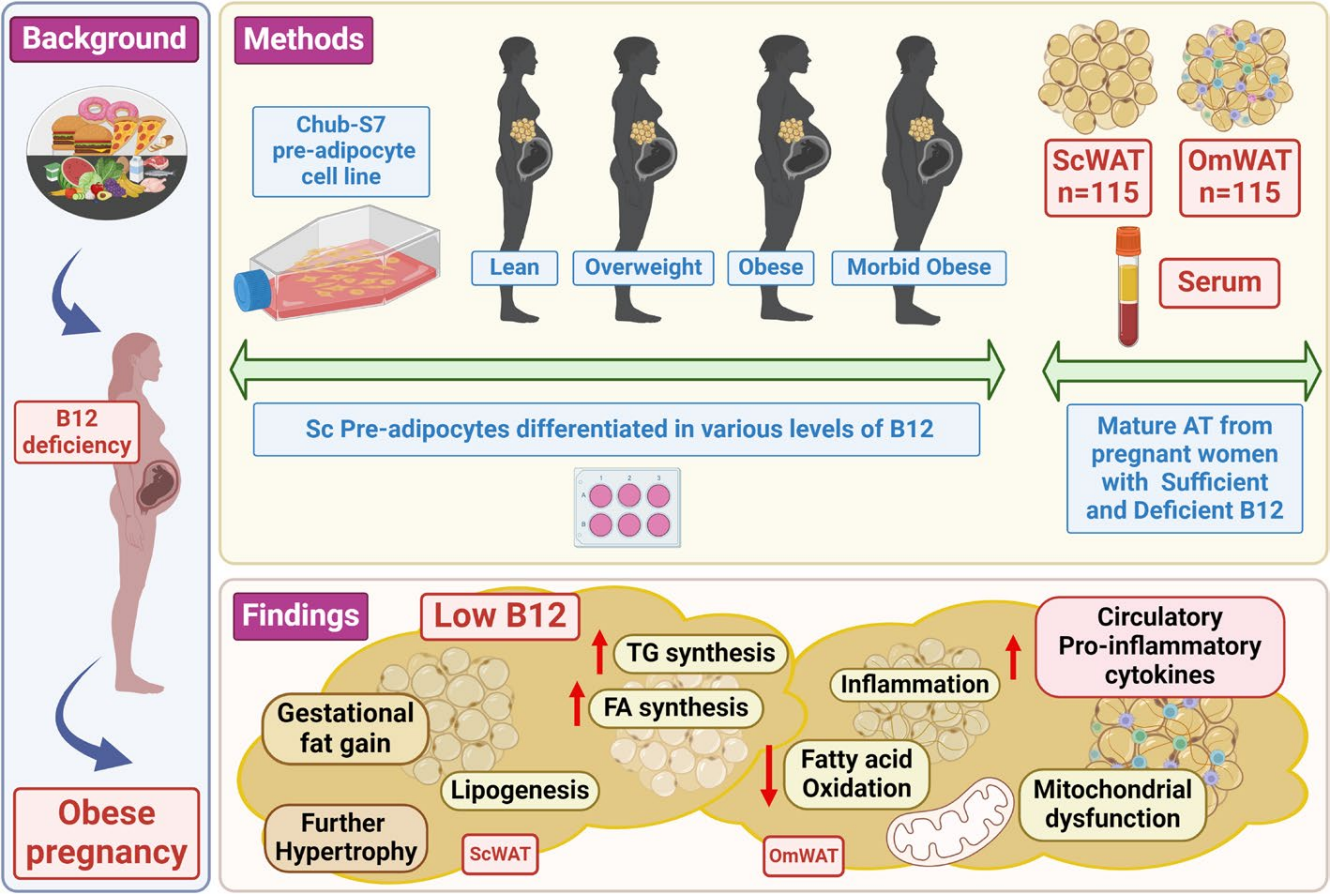

**Supplementary Information:**

The online version contains supplementary material available at 10.1186/s12916-025-04056-4.

## Background

The global prevalence of overweight and obesity in pregnancy is over 60% [1] and maternal obesity is a well-known risk factor for adverse pregnancy outcomes and cardiometabolic risk both in mother and offspring. World Health Organization (WHO) reports that obesity is a double burden of malnutrition resulting from excessive food intake combined with low levels of micronutrients [2–4]. Unfavorable dietary habits including ultra-processed foods, lower consumption of wholegrain cereals, and plant-based diet impact the nutritional status. In the UK, adults are consuming 200–300 extra calories a day and children up to 500 cal extra [2]. Data reports that only 31% of adults consume at least 5 portions of fruit and vegetables a day, and lower proportions are seen in Asian and black adults (19.2% and 21.0%, respectively), and those living in the most deprived areas (20.3%) [4]. While the multifaceted nature of obesity emerges from a complex interaction of genetic factors and environmental influences, it encompasses poor dietary practices. Macronutrients constitute the major food intake, while the level of micronutrients present in this excess calorie intake is notably deficient; moreover, these micronutrients are essential factors in numerous biochemical reactions for the metabolism of macronutrients. This disproportional macronutrient intake results in the conversion of glucose into lipids, such as triglyceride (TG), for storage in the adipose tissue (AT), leading to adipose expansion. Micronutrient malnutrition is a challenge, particularly among women of reproductive age, children, and adolescents, where it contributes to poor metabolic health and to the inter-generational cycle of malnutrition [5, 6].

Studies have evidenced that the micronutrient vitamin B12 (B12) is highly deficient in women at child-bearing age and during the course of pregnancy [7, 8]. B12 deficiency in pregnant women is associated with obesity, gestational diabetes (GDM), and cardiovascular risk [9]. Furthermore, the independent association of TG levels with B12 was shown in pregnant women [9, 10] and patients with type 2 diabetes (T2D) [11]. B12 is the key co-factor for the conversion of homocysteine to methionine, which is the precursor for the synthesis of S-adenosyl methionine (SAM), the methyl donor for methylation reactions. Our prior research studies on adipocytes with low levels of B12 demonstrated hypomethylation of transcription factors (SREBF and LDLR) regulating cholesterol metabolism and alterations of miRNAs (miR- 27, miR- 23, miR- 103, miR- 107) targeting adipogenesis and insulin resistance [12, 13]. In hepatocytes with low B12, we showed dysregulation of genes involved in fatty acid synthesis and oxidation [14, 15]. These studies suggest a pivotal role of B12 in cellular processes such as methylation and other epigenetic regulators of lipid metabolism that may lead to AT dysfunction and development of metabolic diseases. In addition, studies in T2D have reported that deficiency of B12 through the homocysteine pathway might contribute to increased oxidative stress and chronic inflammation [16]. Several other studies have shown association of B12 deficiency with exacerbated inflammation in the brain, vasculature, immune system, liver, and gastrointestinal tract [17–21]. Although obesity and pregnancy independently contribute to a state of low-grade inflammation, the coexistence of obesity during the gestational period seems to exert a synergistic effect, resulting in an elevated level of insulin resistance and a more pronounced inflammatory response, thus increasing the risks of maternal–fetal complications. Moreover, dyslipidemia might trigger an increase in the production of pro-inflammatory cytokines developing into metabolic diseases [22, 23]. Therefore, this study explored the adiposity-related systemic inflammation during pregnancy in the context of B12 deficiency.

We hypothesized that B12 deficiency may affect lipid metabolism as well as mediators of inflammation leading to AT dysfunction. Our aim for this study was to (a) investigate the role of B12 in the regulation of lipid metabolism and inflammation in the human adipocyte cell line (Chub-S7) and human primary pre-adipocytes derived from subcutaneous AT (ScWAT) with different degrees of obesity, (b) validate these findings in depot-specific AT (omental-OmWAT, subcutaneous-ScWAT) from pregnant women with sufficient/deficient levels of B12, and (c) evaluate mitochondrial function and association of B12 with circulatory cytokines and cardiometabolic risk factors.

## Methods

### Human AT study

#### Study subjects

A total of 115 Caucasian pregnant women undergoing elective cesarean section at the University Hospital Coventry and Warwickshire (UHCW) were enrolled for this study. Only non-smoking, non-diabetic, premenopausal women within the ages of 25–38 years and body mass index (BMI) 21.7–41.0 kg/m^2^ were recruited. Ethical approval was sought from the Ethics Committee of UHCW (ID: SK06/9309). All the participants provided written and informed consent in accordance with the Declaration of Helsinki. Anthropometrics including height and weight were recorded for each subject, and venous blood samples were drawn to obtain serum before the cesarean section. Detailed medical drug histories were taken and those participants with cancer, thyroid disorders, or taking steroids or medication or supplements considered to alter inflammatory status including thiazolidinediones were excluded.

#### AT collection

Paired abdominal ScWAT and OmWAT were collected from the consented participants undergoing elective cesarean section. Lean was considered pre-pregnant BMI of less than 25.0 kg/m^2^ and obese was considered pre-pregnant BMI over 30.0 kg/m^2^.

#### Serum cytokine detection with ELISA technique

MCP-1 and IL-8 serum levels were measured by immunosorbent assay (ELISA, R&D Systems, USA) according to the manufacturer’s instructions. The assay and the values given by the manufacturer for intra-assay CV (3.9 ± 0.46%) and inter-assay CV (5.6 ± 0.35%) have been validated in our laboratory.

#### Analytical determinations

Serum B12 and folate were determined by electro-chemiluminescent immunoassay using a Roche Cobas immunoassay analyzer (Roche Diagnostics, UK). We have used 150–660 pM for serum B12 and 6–37 nM for serum folate as normal range [9]. The inter-assay coefficient of variations for B12 and folate were 3.9% and 3.7%, respectively. B12 deficiency was defined as levels below 150 pM. Serum glucose, cholesterol, triglycerides, and HDL cholesterol were determined using an auto analyzer Synchron CX7 (Beckman Coulter, CA) based on enzymatic colorimetric assays. Lipid profiles and fasting plasma glucose were determined using routine laboratory methods at the UHCW NHS Trust. In brief, the routine blood tests included glucose measured by a glucose oxidase method (YSL 200 STAT plus, Yellow Springs Instruments, Yellow Springs, OH, USA) and a standard lipidemic/cholesterol profile (triacylglycerols, HDL cholesterol, and cholesterol). The Friedewald formula was used to compute serum levels of LDL cholesterol [12, 24].

### Cell culture

#### Human preadipocyte Chub-S7 cell line

The Chub-S7 cell line (Nestlé Research Centre, Switzerland) was originally acquired from a pre-adipocyte derived from abdominal ScAT of a 54 kg/m^2^ body mass index (BMI) female, by co-expression of human telomerase reverse transcriptase (hTERT) and papillomavirus E7 oncoprotein (HPV-E7) genes [25]. This cell line is largely used for studies involving adipocyte metabolic function as well as glucose metabolism and uptake, as previously described [26]. The cells uniquely retain the ability to undergo adipogenesis [27]. Proliferating Chub-S7 cells were cultured in growth media (DMEM/Ham’s F- 12 phenol red-free medium 500 ml, 10% fetal bovine serum (FBS), and 1% glutamine) in a T- 75 flask until 90% confluence. At this stage, the cells were seeded onto 6-well plates at a density of 30,000 cells per well and grown to confluence (day 0), differentiated in differentiation media (DMEM/Ham’s F- 12 phenol free medium 500 ml, 3% FBS, and Promocell preadipocyte differentiation medium supplement (Promocell cat#C39436) mix: 0.5 µg/ml human insulin, 400 ng/ml dexamethasone, 8 µg/ml d-biotin, 44 µg/ml IBMX, 9 ng/ml L-thyroxine, 3 µg/ml ciglitazone) for 7 days and maintained in nutrition media (Promocell cat#C39439) for the next 7 days (day 14). To analyze B12 deficiency effects, customized DMEM/Ham’s F- 12 media (In-house media preparation facility, Life sciences, University of Warwick) with different B12 concentrations (500 nM, 1 nM, 100 pM, 25 pM) were used. On day 14, the cells were harvested using 500 µl of Qiazol (Qiagen cat#79306) for RNA analysis and stored at − 80 °C until required.

#### Human primary preadipocytes

Stromal vascular cell isolation was performed on ScAT collected from non-smoking, non-diabetic, premenopausal, Caucasian women with different BMI (subjects with lean, overweight, obesity, and morbid obesity) undergoing elective cesarean section. The white adipose tissue (WAT) biopsy was digested in collagenase at 37 °C in a shaking water bath. The resulting cell suspension was then centrifuged at 2000 rpm for 5 min. The obtained stromal vascular fraction was re-suspended in primary adipocyte growth media (DMEM/Ham’s F- 12 phenol-free medium 500 ml (Invitrogen-cat#11,039,047), 10% fetal bovine serum, 1% glutamine, fibroblast growth factor-basic 5 ng/ml (cat#VXPHG0026), and transferrin 5 µg/ml (cat#VX0030124SA)) and transferred to a 75-cm^2^ tissue culture flask. The flask was maintained in a 37 °C, 5% CO_2_ humidified incubator and the growth media was changed every 2 days until 90% confluence was reached. At this stage, the cells were seeded into 6-well plates at a density of 30,000 cells per well and grown to confluence (day 0), differentiated in differentiation media for 7 days and maintained in nutrition media for an additional 7 days (day 14). To analyze B12 deficiency effects, the primary adipocytes (*n* = 3 per BMI group with 6 replicates from each group) derived from subcutaneous depots were seeded and differentiated in customized media with concentrations of B12 as control (500 nM) and low B12 (25 pM). On day 14, the cells were harvested using 500 µl of Qiazol for RNA analysis and stored at − 80 °C until required.

### RNA isolation, cDNA synthesis and gene expression assay

RNA extraction from primary adipocytes and Chub-S7 was carried out using Qiazol, and from AT samples using an RNeasy lipid tissue kit (Qiagen, UK) according to the manufacturer’s instructions followed by a DNase digestion step, as previously described [14, 24]. RNA quantification, quality, and integrity (260/230 and 260/280 ratios and concentrations) were carried out using a NanoDrop spectrophotometer (Labtech, UK) and 300 ng was used for cDNA synthesis as described previously [14]. The gene expression assays were performed using qRT-PCR and the housekeeping genes, 18s rRNA was used for Chub-S7 cell line (cat no 4319413E; Applied Biosystems, Thermo Fisher Scientific, UK) and L19 for AT (forward primer—GGAAAAAGAAGGTCTGGTTGGA, reverse primer—TGATCTGCTGACGGGAGTTG; Merck Life Science UK Ltd, Dorset, UK) were used to normalize and determine relative gene expression.

### Radiochemical flux assessment of TG synthesized via utilization of radiolabeled fatty acid (^14^C-oleate) by Chub-S7 and primary adipocytes

The amount of TG synthesized by cells over a period of time was quantified by initially exposing cells to radiolabeled fatty acid (^14^C-oleate) in the presence of fatty acid co-transporter (L-carnitine) as previously described [14, 28]. The radiolabeled TG synthesized was extracted on a thin layer chromatography plate with the inclusion of glyceryltripalmitate as the standard. This was quantified with a scintillation counter (Beckman Coulter LS6500, USA) and normalized to protein content obtained via Bradford assay [29].

### Mitochondrial function assessment using seahorse extracellular flux assay

#### Cell culture for seahorse assay

Oxygen consumption rates (OCR) was measured using a Seahorse XF24 Extracellular Flux Analyzer (Seahorse Bioscience, Santa Clara, CA, USA). Chub-S7 and primary adipocytes were trypsinized and seeded in 24-well (XF- 24) seahorse plates at a density of 10,000 cells/well under different B12 concentrations of EMEM media supplemented with 10% FBS, 1% L-glutamine, and 1% streptomycin/penicillin and incubated at 37 °C with 5% CO_2_ saturation. The respective B12 media were changed every 48 h until reaching 90–100% confluence.

On the day of the Seahorse XF experiment, Chub-S7 and primary adipocytes were washed in Krebs–Henseleit Buffer (KHB) by removing the media from the seahorse culture plates followed by addition of 1 ml of KHB. Then, the KHB was removed, followed by a final addition of another 675 μl of KHB to each well of the plate. The plates were then incubated at 37 °C for 1 h without CO_2_ saturation. To run the seahorse assay, the cartridge and the seahorse XF24 plate were loaded into the seahorse analyzer for measurement of OCRs as previously described [14, 28]. The 10 × concentration (stock) of the inhibitors were optimized and prepared in KHB buffer to obtain final concentrations (1 ×) of oligomycin (0.4 μM), carbonyl cyanide-p-tri-fluoro-methoxy-phenylhydrazone (FCCP—0.75 μM), and rotenone/antimycin (0.4 μM) after injection into seahorse XF24 plates. The inhibitors were loaded into the allocated ports of the seahorse cartridge which was initially hydrated in 1 ml calibrant for 24 h at 37 °C without CO_2_ saturation.

#### Maximal respiratory capacity

Briefly, the basal oxygen consumption rate (OCR) measurement was performed in Chub-S7 and primary adipocytes in a rich substrate media (glucose—2.5 mM, pyruvate—1 mM, L-glutamine—2 mM, BSA—0.1%) by addition of the inhibitors oligomycin and FCCP followed by antimycin/rotenone using Seahorse 24XF flux analyzer [24, 28].

#### Respiratory capacity in a limited substrate (high palmitate) supply

To examine how the low B12 adipocytes function with the endogenous supply of high extracellular levels of palmitate and other limited substrate, we incubated Chub-S7 and primary adipocytes in a limited-substrate KHB medium (L-carnitine—0.5 mM and glucose—1.25 mM), which is poorly enriched with other supplements compared with the rich-substrate KHB medium for 1 h. Firstly, the basal OCR was measured, then the cells were either exposed to 200 μM palmitate (dissolved in 33.3 μM BSA) or 33.3 μM BSA only (basal control) in the limited substrate medium was measured to assess how adipocytes efficiently uptake palmitate for ATP metabolism.

### Statistical analysis

The data was expressed as mean ± standard error of mean (SEM). All statistical analyses were performed using SPSS 24.0 (Statistical Package for the Social Sciences, Chicago, USA) or Prism 8 (GraphPad, San Diego, USA). Kolmogorov–Smirnov’s and Shapiro–Wilk test were used to determine the parametric distribution of data. Data obtained from the scintillation count of the radioactive flux assay were normalized with the total protein concentration (mg) in each sample (*n*) of Chub-S7 and adipocytes. The Mann–Whitney *U* test was used for comparing two groups of non-normally distributed data, while the two-tailed Student’s *t*-test was applied for comparison of normally distributed data. Correlations were assessed using Spearman’s method for non-normally distributed parameters. *p* value of < 0.05 was considered statistically significant.

## Results

### Clinical and biochemical characteristics of participants

Among the participants (*n* = 115) recruited in the study, more than half (*n* = 63) had sufficient levels of B12 (> 150 pM), whereas the remaining participants (*n* = 52) had deficient levels of B12 (< 150 pM). The clinical characteristics and biochemical data are presented in Table 1. Assessment of anthropometric data showed that BMI was significantly higher in women with deficient B12 levels than women with sufficient B12 levels (B12 deficient group vs B12 sufficient group: 28.5 vs 26.5 kg/m^2^). In addition, in the B12 deficient group, biochemical parameters such as folate (22.4 vs 14.9 nM) and HDL cholesterol (1.4 vs 1.7 mM) levels were significantly lower, whereas TG (3.5 vs 2.9 mM) was higher. However, other biochemical parameters (glucose, total cholesterol, LDL) were not statistically significant between the groups.

**Table 1:** Clinical and biochemical characteristics according to vitamin B12 levels

Variables	B12 sufficient group (> 150 pM) *n* = 63	B12 deficient group (< 150 pM) *n* = 52	*p* value
Age (years)	32.1 ± 5.7	31.5 ± 6.5	0.296
Weight (kg)	73.2 ± 14.6	75.6 ± 19.4	0.708
BMI (kg/m^2^)	26.5 ± 3.9	28.5 ± 6.37	**0.041**
Glucose (mM)	3.68 ± 0.6	3.71 ± 0.6	0.787
Cholesterol (mM)	6.6 ± 1.4	6.7 ± 1.5	0.947
HDL (mM)	1.7 ± 0.4	1.4 ± 0.2	**0.0002**
LDL (mM)	4.3 ± 1.2	4.8 ± 1.6	0.722
Triglycerides (mM)	2.9 ± 0.9	3.5 ± 1.0	**0.0094**
Folate (nM)	22.4 ± 12.2	14.9 ± 9.7	**0.0009**

The data is expressed as mean ± SD. *p* values were obtained using the Mann–Whitney *U* test; *p*-value < 0.05 was considered significant

*Abbreviations:* *SD* standard deviation, *BMI* body mass index, *HDL* high-density lipoprotein, *LDL* low-density lipoprotein

### Low B12 increases lipogenesis in human Chub-S7 and primary Sc adipocytes

**Fig. 1:**
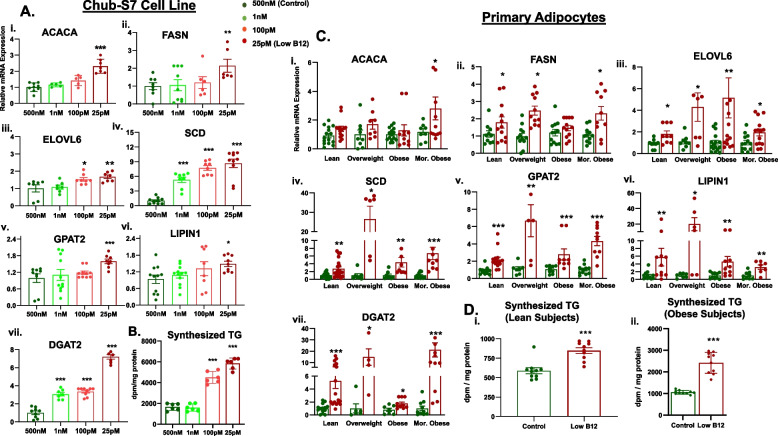
Effect of low B12 on lipogenesis in human Chub-S7 cell line and primary Sc adipocytes: [**A**] and [**C**] Expression of genes involved in FA synthesis (ACACA, FAS), elongation (ELOVL6), desaturation (SCD) and TG synthesis (GPAT2, LIPIN1, DGAT2) in human (i-vii) Chub-S7 cell line (*n*=6) and (i-vi) primary Sc adipocytes (n=3 per BMI group - subjects with lean, overweight, obesity, morbid obesity), respectively. The Chub-S7 cells were seeded and differentiated with different concentrations of B12 (500nM, 1nM, 100pM, 25pM), and gene expression were measured by RT-qPCR and normalized to 18s rRNA. The primary adipocytes were derived from subcutaneous depots, which were seeded and differentiated with two different concentrations of B12 (500nM, 25pM). The gene expressions normalized to L19 were measured by RT-qPCR. Levels of synthesized TG assessed by radiochemical flux assay in [**B**] Chub-S7 adipocytes, [**D**] Primary Sc adipocytes derived from subjects with (i) Lean BMI and (ii) Obesity. The cells were first labelled with ^14^C-Oleate for 2 h, then followed by total lipids extraction and the resultant radiolabelled TG was separated on a thin layer chromatography (TLC) plate with glyceryltripalmitate as standard, quantified with the scintillation counter (Beckman coulter LS6500, USA) and normalized per milligram protein estimated with Bradford method. Dots depict individual study subjects and data represented as mean ± SEM (standard error of mean) and significance levels indicated as follows; *p<0.05, **p<0.01 compared to control B12 (500nM)

The study investigated the effect of low B12 on de novo lipogenesis in Chub-S7 cells and primary Sc adipocytes cultured in different concentrations of B12. We observed that the expression of FA synthesis (ACACA, FASN, ELOVL6) and TG synthesis (SCD, GPAT2, LIPIN1, DGAT2) genes were significantly higher in low B12 Chub-S7 adipocytes compared to control (Fig. 1A, i–vii, *p* < 0.05). In addition, we observed an upregulation of 10–30-fold for SCD, LIPIN1, and DGAT2 genes in low B12 primary Sc adipocytes derived from subjects with overweight and an increase of 20-fold for DGAT2 from subjects with morbid obesity (Fig. 1C, i–vi, *p* < 0.05). Following the observation of upregulation of key genes in lipogenesis, we further assessed how this would affect the utilization of radiolabeled FAs by adipocytes for TG biosynthesis. We performed the radiochemical analysis by exposing the cells to radiolabeled FA (^14^C-oleate) and assessed the amount of radiolabeled TG synthesized. We demonstrated that the levels of radiolabeled TGs synthesized in Chub-S7 cells was increased 2.7–3.6-fold in low B12 (Fig. 1B, *p* < 0.05). Primary Sc adipocytes derived from subjects with lean BMI had significantly 1.5-fold higher TG synthesis whereas subjects with obesity had 2.5-fold (Fig. 1D, i–ii, *p* < 0.05) higher in the low B12 condition. These findings indicate that not only were adipocytes from obese phenotype were more prone to lipid accumulation, but adipocytes from lean individuals also displayed increased lipid content, when they were subjected to B12 deficiency.

### B12 deficiency upregulates lipogenesis in mature human WAT

**Fig. 2:**
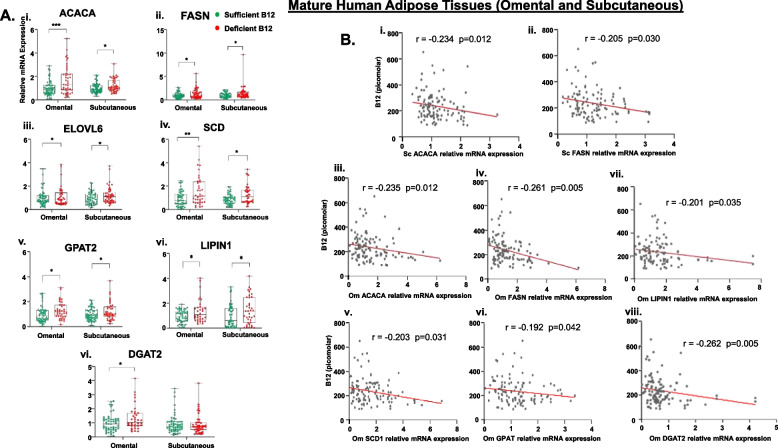
Effect of B12 deficiency on lipogenesis in mature human AT (ScWAT and OmWAT) and its correlation with serum B12. [**A**] Expression of genes involved in FA synthesis (i-vii) (ACACA, FAS), elongation (ELOVL6), desaturation (SCD) and TG synthesis (GPAT2, LIPIN1 and DGAT2) in ScWAT and OmWAT. Total - 115 pregnant women, subjects with sufficient levels of B12 (>150pM) = 63 (green) and Deficient levels of B12 (150pM) = 52 (red)). The gene expression involved in FA and TG biosynthesis was assessed using RT-qPCR and normalized to L19. The data are reported as box charts median (IQR) and significance levels are indicated as *p<0.05, **p<0.01 compared to sufficient B12 (>150pM). [**B**] (i-ii) Correlation between Sc ACACA and FASN gene expression with serum B12; (iii-viii) Correlation between Om ACACA, FASN, SCD1, GPAT, LIPIN1, DGAT2 gene expression with serum B12. Dots depict individual study subjects and data are presented as Spearman’s R-values: ACACA: Acetyl-CoA Carboxylase Alpha, FASN: Fatty Acid Synthase, ELOVL6: Elongation of very long Fatty Acid Elongase 6, SCD: Stearoyl- CoA Desaturase, GPAT2: Glycerol-3-Phosphate Acyltransferase 2, Mitochondrial, LIPIN1 Phosphatidate Phosphatase, and DGAT2: Diacylglycerol O-Acyltransferase 2

To validate the evidence of low B12 effect on lipogenesis in the Chub-S7 cells and primary Sc adipocytes, gene expression was further investigated in ScWAT and OmWAT derived from pregnant women with sufficient and deficient levels of B12. Our findings revealed that B12 deficient women had significant increase in expression of genes involved in the de novo FA synthesis (ACACA, FASN), elongation (ELOVL6, SCD), and TG biosynthesis pathways (GPAT2, LIPIN1) in both OmWAT and ScWAT compared to subjects with adequate B12 levels. Although there was a significant upregulation of DGAT2 gene expression in OmWAT of B12 deficient women, no difference was observed in the ScWAT (Fig. 2A, i–vii, *p* < 0.05).

### Serum B12 correlates with Sc and Om lipogenesis

To assess the association of lipogenesis in mature human AT with serum levels of B12 and the association with serum biochemical metabolites, we performed a correlation analysis. In ScWAT, the data showed a significant negative correlation between serum B12 and the expression of FA genes including ACACA and FASN (Fig. 2B, i–ii, *p* < 0.05). In OmWAT, there was a significant negative correlation between B12 and gene expression of ACACA, FASN, SCD, GPAT2, LIPIN1, and DGAT2 (Fig. 2B, iii–viii, *p* < 0.05).

There was a significant positive correlation between gene expression of ScACACA and serum total cholesterol (*r* = 0.201, *p* = 0.036), LDL cholesterol (*r* = 0.221, *p* = 0.031), and TGs (*r* = 0.211, *p* = 0.031). Furthermore, a significant positive correlation was observed between ScGPAT2 and glucose (*r* = 0.222, *p* = 0.019) (Additional file 1: Table 1). Likewise, there was a significant negative correlation between OmACACA and serum levels of HDL (*r* = − 0.209, *p* = 0.040). Moreover, a significant positive correlation between OmFASN and serum TG (*r* = 0.220, *p* = 0.025) was observed, whereas OmELOVL6 significantly correlated positively with serum total cholesterol (*r* = 0.247, *p* = 0.009), LDL (*r* = 0.298, *p* = 0.002), and TGs (*r* = 0.210, *p* = 0.027). Additionally, OmSCD positively correlated with serum TG (*r* = 0.220, *p* = 0.022), whereas OmLIPIN1 negatively correlated with HDL (*r* = − 0.217, *p* = 0.036), and OmDGAT2 positively correlated with TG (*r* = 0.194, *p* = 0.044) (Additional file 1: Table 2). These data suggest that there was strong negative correlation between B12, lipid synthesis genes, and lipid profile in OmWAT than ScWAT.

### Low B12 impairs FA β-oxidation and mitochondrial function in human Chub-S7 and primary Sc adipocytes

**Fig. 3:**
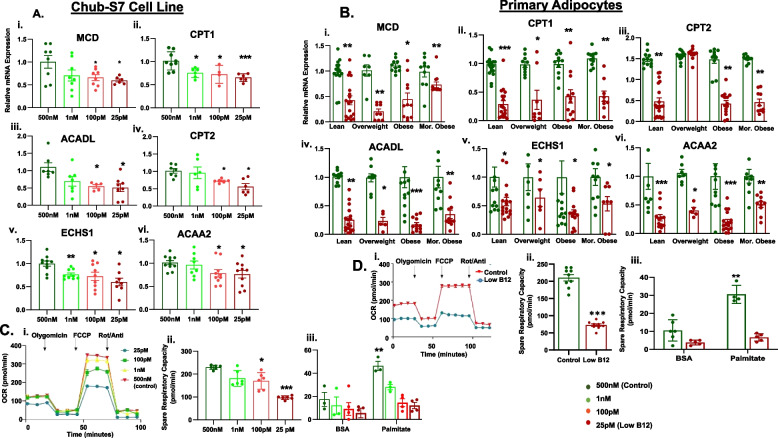
Effect of low B12 on FA β-oxidation and mitochondrial function in Chub-S7 cell line and primary Sc-adipocytes. [**A**] and [**B**] Expression of genes (MCD, CPT1, ACADL, CPT2, ECHS1, ACAA2) involved in FA β-oxidation in (i-vi) Chub-S7 cell line (*n*=6) and (i-vi) primary Sc-adipocytes (*n*=3 per BMI group - subjects with lean, overweight, obesity, morbid obesity), respectively. The Chub-S7 cells were seeded and differentiated with different concentrations of B12 (500nM, 1nM, 100pM, 25pM), and gene expression were measured by RT-qPCR and normalized to 18s rRNA. The primary adipocytes were derived from subcutaneous depots, which were seeded and differentiated with two different concentrations of B12 (500nM, 25pM). The gene expressions normalized to L19 were measured by RT-qPCR. The graphs in [**C**] Chub-S7 and [**D**] primary Sc-adipocytes, respectively, show the (i) Oxygen consumption rate (OCR) and (ii) Spare respiratory capacity in cells incubated with a rich-substrate KHB media (containing glucose - 2.5mM, pyruvate - 1mM, L-Glutamine - 2mM, BSA - 0.1%) at pH 7.4 under various B12 conditions. (iii) Represents the spare respiratory capacity following exposure with palmitate (200µM)/ Basal control (BSA - 33.3µM) in a limited-substrate KHB media (containing only L-carnitine - 0.5mM, glucose - 1.25mM) under various conditions of B12. Dots depict individual study subjects and data represented as mean ± SEM and significance levels indicated as *p<0.05, **p<0.01, ***p<0.001, compared to control B12 (500nM)

Along with increased lipogenesis, we further wanted to examine the expression of genes required for mitochondrial FA transport and β-oxidation of FAs in low B12 condition. In the Chub-S7 cells and primary Sc adipocytes from all metabolic phenotypes including lean, overweight, obese, and morbid obese, we found a significant reduction in the expression of FAO genes including MCD, CPT1, ACADL, CPT2, ECHS1, and ACAA2 in low B12 (Fig. 3A, i–vi; B, i–vi, *p* < 0.05). Then, we analyzed the mitochondria functional integrity in both the in vitro/ex vivo models, where we observed an overall decrease of oxygen consumption rate (OCR) in low B12 (Fig. 3C, i; D, i, *p* < 0.05). We further analyzed the spare respiratory capacity (SRC), a measure of the ability of the cells to respond to increased energy demand in a limited substrate supply. The SRC was significantly reduced in low B12 Chub-S7 cells, when supplied either with a substrate-rich medium with high glucose (Fig. 3C, ii) or a substrate-limited medium, low glucose supplemented with palmitate (Fig. 3C, iii). This implies that in low B12, FA (palmitate) utilization for energy metabolism by the mitochondria is significantly reduced. Similar results were also observed in the primary Sc adipocytes in which low B12 cells had a significantly decreased SRC, when supplemented with a substrate-rich medium (Fig. 3D, ii) as well as a substrate-limited medium with high palmitate (Fig. 3D, iii) (*p* < 0.05).

### B12 deficiency impairs FA β-oxidation in mature human ATs and its association with serum B12

**Fig. 4:**
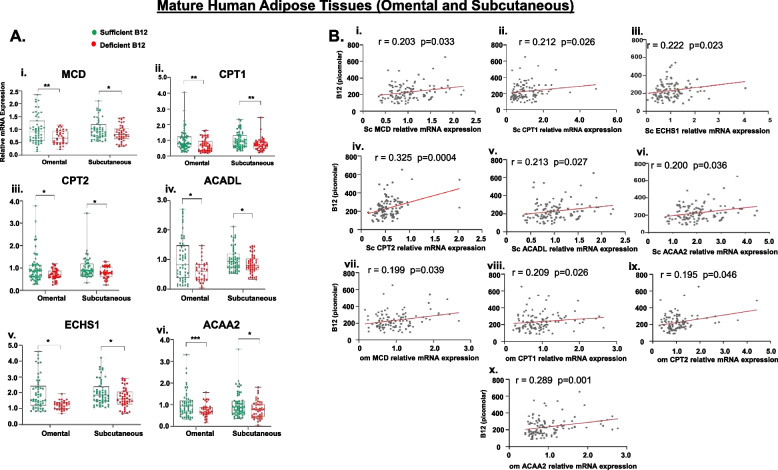
Effect of B12 deficiency on FA β-oxidation in mature human AT (omWAT and scWAT), and its association with serum B12: [**A**] Expression of genes (i-vi) (MCD, CPT1, CPT2, ACADL, ECHS1, ACAA2) involved in FA β-oxidation in omWAT and scWAT. Total - 115 pregnant women, subjects with sufficient levels of B12 (>150pM) = 63 (green) and Deficient levels of B12 (<150pM) = 52 (red)). Data presented as box charts median (IQR). Significance levels are indicated as *p<0.05, **p<0.01, ***p<0.001 compared to Sufficient B12 (>150pM). [**B**] (i-vi) Correlation between Sc-FA oxidation genes MCD, CPT1β, ECHS1, CPT2, ACADL, ACAA2, respectively with serum B12, and (vii-x) correlation between Om-FA oxidation genes MCD, CPT1β, CPT2, ACAA2 respectively with serum B12. The dots depict individual study subjects and data presented as Spearman’s R-values. MCD: Malonyl-CoA decarboxylase, CPT1β: Carnitine palmitoyltransferase-1, CPT2: Carnitine palmitoyltransferase-2, ACADL: Acyl-CoA Dehydrogenase Long Chain, ECHS1: Enoyl-CoA Hydratase, Short Chain 1 and ACAA2: Acetyl-CoA Acyltransferase

Similar to lipogenesis, we further validated the expression of genes involved in FAO in AT. The relative expression of FAO genes (MCD, CPT1β, CPT2, ACADL, ECHS1, ACAA2) was significantly decreased in both OmWAT and ScWAT of B12 deficient women compared with subjects with sufficient levels of B12 (Fig. 4A, i–vi, *p* < 0.05). Correlation analysis was performed to understand the relationship between B12, biochemical metabolites, and FAO gene expression in AT. In the ScWAT, a positive correlation was observed between serum B12 levels and FAO genes (MCD, CPT1β, ECHS1, CPT2, ACADL, ACAA2, Fig. 4B, i–vi). In the OmWAT, there was a positive association between serum B12 and FAO (MCD, CPT1β, CPT2, ACAA2, Fig. 4B, vii–x, *p* < 0.05).

Serum metabolites in the ScWAT showed a positive correlation between HDL and ScCPT2 (*r* = 0.231, *p* = 0.015) and ScECHS1 (*r* = 0.192, *p* = 0.044), whereas in the omWAT, there was a negative correlation between glucose and ScCPT2 (*r* = 0.202, *p* = 0.05), TG and ScCPT2 (*r* = 0.217, *p* = 0.04), and TG and ScACAA2 (*r* = 0.215, *p* = 0.044) (Additional file 1: Tables 3 and 4). These findings affirm that FAO genes in AT associated with adverse circulating lipids, which was further exacerbated in low B12 status.

### Low B12 induces inflammatory cytokines in human Chub-S7 and primary Sc adipocytes

**Fig. 5:**
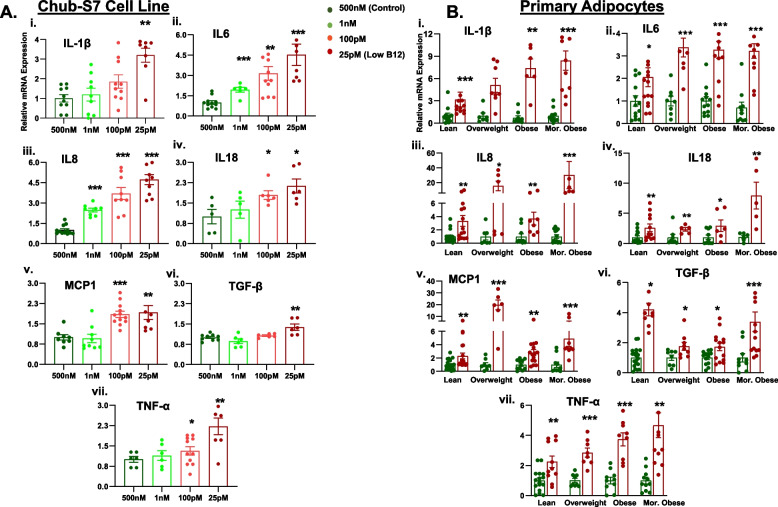
Effect of low B12 on inflammatory cytokines in Chub-S7 cell line and primary Sc-adipocytes [**A**] and [**B**] Expression of genes involved in inflammation (IL-1β, IL-6, IL-8, IL-18, MCP-1, TGF-β and TNF-α) in (i-vii) Chub-S7 cell line (*n*=6) and (i-vii) primary Sc-adipocytes (*n*=3 per BMI group - subjects with lean, overweight, obesity, morbid obesity), respectively. The Chub-S7 cells were seeded and differentiated with different concentrations of B12 (500nM, 1nM, 100pM, 25pM), and gene expression were measured by RT-qPCR and normalized to 18s rRNA. The primary adipocytes were derived from subcutaneous depots, which were seeded and differentiated with two different concentrations of B12 (500nM, 25pM). The gene expression normalized to L19 were measured by RT-qPCR. Dots depict individual study subjects and data expressed as mean ± SEM and significance levels indicated as follows; *p<0.05, **p<0.01, **p<0.001 compared to control B12 (500nM)

**Fig. 6:**
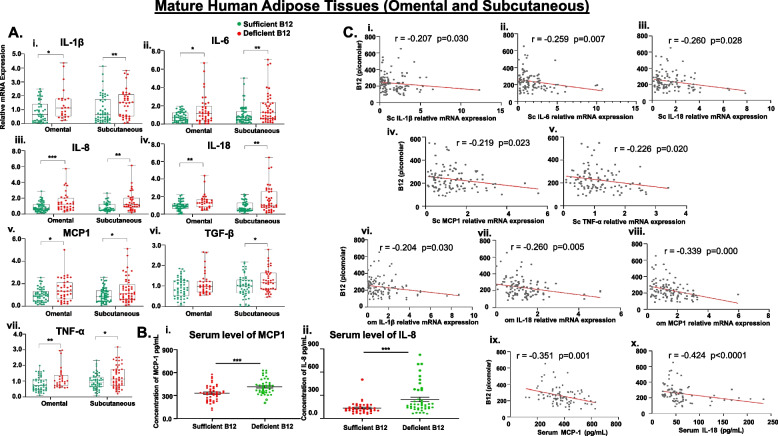
Effect of low B12 on inflammatory cytokines in mature human AT, and its association with circulating B12 and biochemical metabolites [**A**] Expression of genes involved in inflammation (i-vii) IL-1β, IL-6, IL-8, IL-18, MCP-1, TGF-β and TNF-α in OmWAT and ScWAT. Total - 115 pregnant women, subjects with sufficient levels of B12 (>150pM) = 63 (green) and Deficient levels of B12 (<150pM) = 52 (red)). The data are not normally distributed and reported as box charts median (IQR). Significance levels are indicated as follows *p<0.05, **p<0.01, ***p<0.001 compared to Sufficient B12 (>150pM). [**B**] Circulating levels of (i) MCP1 and (ii) IL-8 levels in serum. The dots depict individual study subjects, the line represents the mean, and the whiskers depict the SEM. [**C**] (i-v) Correlation between gene expression of Sc inflammatory markers IL-1β, IL-6, IL-18, MCP-1, TNFα and serum B12. (vi-viii) Correlation between Om-inflammatory markers (IL-1β, IL-18, MCP-1) and serum B12, respectively. (ix-x) Correlation between circulating levels of MCP-1 and IL-8 and serum B12, respectively. Dots depict individual study subjects and data presented as Spearman’s R-values. IL-1β: interleukin-1 beta, IL-6: interleukin-6, IL-18: interleukin-18; MCP- 1: monocyte chemoattractant protein-1; TNFα: Tumour necrosis factor-α

To further explore whether inflammation is a possible mechanism mediating the impact of B12, we next examined the pro-inflammatory cytokines. Chub-S7 cells cultured in low B12 showed significantly increased pro-inflammatory cytokine gene expression such as IL- 1β, IL- 6, IL- 8, IL- 18, MCP- 1, TGF-β, and TNF-α compared to control (Fig. 5A, i–vii, *p* < 0.05). Our findings also revealed that adipocytes from all the categories of metabolic phenotype showed a significant upregulation in B12 deficient culture. Interestingly, we observed that Sc adipocytes derived from overweight subjects showed an increase of 15-fold for IL- 8 and 20-fold for MCP- 1, and adipocytes from subjects with morbid obesity showed an increase of 30-fold for IL- 8 and eightfold for IL- 18 gene expression in low B12 (Fig. 5B, i–vii, *p* < 0.05). Furthermore, both ScWAT and OmWAT revealed a significant increase in the expression of inflammatory cytokine genes in B12 deficient subjects compared with subjects with adequate levels of B12 (Fig. 6A, i–vii). Since MCP1 and IL- 8 showed several folds of upregulation in isolated Sc adipocytes and both depots of WATs, we investigated the serum levels of MCP1 and IL- 8 pro-inflammatory cytokines in low B12 women. We found a significantly increased levels of MCP1 and IL- 8 in the serum of B12 deficient women compared to those with sufficient levels of B12 (Fig. 6B, i–ii, *p* < 0.05). These results indicate that B12 deficiency affects the levels of the cytokines in AT and circulation, thus inducing pro-inflammatory factors which could contribute to adipose dysfunction.

### Association between inflammatory cytokines in ATs, circulating B12 and biochemical metabolites

In the ScWAT, correlation analysis showed a significant negative association between serum B12 and gene expression of IL- 1β, IL- 6, IL- 18, MCP- 1, and TNF-α (*p* < 0.05), but not with IL- 8 and TNFβ (Fig. 6C, i–v). In OmWAT, a significant negative association was shown between serum B12 and gene expression of IL- 1β, IL- 18, and MCP- 1 (Fig. 6C, vi–viii, *p* < 0.05). Serum levels of MCP- 1 and IL- 8 significantly correlated negatively with serum B12 (Fig. 6C, ix–x, *p* < 0.05).

Next with serum metabolites, we observed a significant positive correlation of ScIL- 1β with LDL, MCP- 1, and IL- 8. A positive correlation of ScIL- 8 with glucose and triglycerides but negatively with HDL was observed. However, ScMCP- 1 negatively correlated with serum HDL (Additional file 1: Table 5). In addition, OmIL- 6 positively correlated with LDL and negatively with serum IL- 8, whereas OmIL- 8 negatively correlated with glucose and HDL. Similarly, OmIL- 18 negatively correlated with HDL but positively with TG and serum IL- 8. In addition, OmTGF-β negatively correlated with cholesterol and LDL whereas OmTNFα positively correlated with glucose and HDL (Additional file 1: Table 6). Taken together, these findings therefore confirmed that any differences that we identified in inflammatory gene expression in AT were related to any changes in circulating lipids thereby suggesting an early inflammatory AT phenotype may be a mechanism that underlies the development of metabolic disorders as a consequence of low B12 levels in maternal obesity.

## Discussion

The study provides the comprehensive evaluation of in vitro and ex vivo models along with sc/omWAT in the context of inflammation in response to obesity and micronutrient deficiency. We demonstrated that pregnant women with B12 deficiency were obese and had dyslipidemia with altered inflammatory cytokines. This was accompanied by adverse transcriptional changes in OmWAT and ScWAT including increased FA synthesis and inflammation. In addition, in vitro AT analysis revealed impaired FAO and mitochondrial function not only in obese adipocytes but also pronounced changes in lean adipocytes when subjected to low B12. These differences are indicative of increased lipid synthesis and enhanced inflammation in individuals with B12 deficiency which may contribute to adipose dysfunction often observed in obesity.

Increased obesity is a common comorbidity of metabolic syndrome associated with T2D and cardiometabolic risk. Clinical correlations revealed that B12 was associated with FA synthesis genes in both depots of WAT and the lipid profile, but more distinct in OmWAT. This highlights that AT is prone to increased fat storage not only when there are excess glucose or lipids, but when the environment is deficient in micronutrients such as B12. Accordingly, we studied the gene expression in obese and lean human primary adipocytes in vitro exposed to low B12. In assessing lipogenesis, we found there was significant upregulation of genes involving FA and TG synthesis in low B12 not only in adipocytes from obese phenotype but also in adipocytes from lean subjects. Our previous studies have reported similar evidence in hepatocytes with low B12 demonstrating upregulation of FA and TG synthesis [14], and increased adiposity via hypomethylation and upregulation of the master regulators of lipogenesis (SREBF1) and cholesterologenesis (LDLR) in adipocytes [13]. A recent study in *Caenorhabditis elegans* revealed that early-life B12 deficiency caused increase in the levels of fat and demonstrated that the methionine cycle-SBP- 1/SREBP1-lipogenesis axis was required for early-life B12 to determine health outcomes later in life [30]. Furthermore, it is reported that supplementation of B12 at a critical developmental time window rescued the fat phenotype, particularly at early stages. However, the programmed disorders at later life were not effective with supplementation of B12 but reversed through suppression of SBP- 1/SREBP1-lipogenesis signaling. Since simultaneous upregulation of FAO rate and mitochondrial health is a mechanism to augment the release of FA from AT [31], our study further investigated the expression of FAO genes in low B12 adipocytes and mature human sc/omWAT and found they were significantly impaired. Reduction in FAO capacity has been reported with obesity and animal models with restricted B12 [32]. Furthermore, the incidence of metabolic disorders affecting tissues such as the liver, heart, and skeletal muscles that solely depend on FAO for metabolism has been reported [33]. Previous studies in hepatocytes also reported a significant reduction in the rate-limiting enzyme CPT1α and downstream genes involved in FAO as a result of low B12 [14]. Conversely, models with supplementation of deprived mice with methyl donors, such as B12 and folate, showed alleviation of hepatic fatty infiltration via upregulation of FAO [34]. This was achieved following a significant reduction in carnitine levels signifying an upregulation in CPT1α [35]. To confirm our observation of low B12-induced FAO impairment in AT, we assessed mitochondria functional integrity in a preadipocyte cell line and primary adipocytes, and the correlation of B12 with FAO genes in mature human scWAT and omWAT. Low B12 resulted in a significant reduction in OCR and SRC suggesting an impaired mitochondrial function, whereas a significant positive correlation observed between serum B12 and FAO genes in scWAT and omWAT affirmed the changes. Our evidence supports that B12 deficiency induces dysregulation of lipid metabolism promoting synthesis and storage of FAs in AT with reduced FA catabolism and mitochondrial dysfunction. As such, if AT is not able to buffer lipids in this low B12 induced state, lipids will spill over into the bloodstream resulting in dyslipidemia, an underlying cause of obesity-associated inflammation and IR.

In addition to lipid dysregulation and mitochondrial dysfunction, we found that serum B12 negatively correlated with inflammatory genes (IL1β, IL6, IL8, IL18, MCP1, TGFβ, and TNFα) in both depots of WAT. We also observed increased expression of pro-inflammatory genes in both in vitro and ex vivo models of adipocytes. IL8, IL18, and MCP1 were several folds higher in obese adipocytes with low B12 than lean adipocytes. Further, the circulating levels of inflammatory cytokines (MCP1, IL- 8) were associated with low B12 and lipid profile. These findings highlight that the pro-inflammatory responses in AT are intrinsically linked with levels of B12. Previous clinical studies showed B12 deficiency is associated with inflammation and cardiometabolic risk and revealed that serum B12 is associated with inflammatory molecules (IL- 6, CRP) [36]. In a cross-sectional study with middle-aged participants, a negative association was observed between TNF-α and serum B12 [37]. A recent study in Crone’s disease showed pro-inflammatory cytokines IL- 1β and TNF-α were significantly upregulated during folate and vitamin B_12_ deprivation following *Mycobacterium avium paratuberculosis* infection [38]. Further to this, data from naturally aged, healthy wild-type mice provides supporting evidence by also showing an inverse relationship between serum B12 and IL- 6. Rats fed with B12 deficient diet triggered NASH through activation of pro-inflammatory pathways with an imbalanced release of IL- 1β, IL- 10, and MCP1 [19]. Supplementation studies with both humans and animals demonstrate that B12 may have a positive effect on inflammatory status via reduction of hyperhomocysteinemia or improving the antioxidant capacity [39, 40]. To our knowledge, this is the first study showing results in human primary adipocytes and different depots of AT with varying levels of B12. Our data supports an important role of B12 on inflammatory secretion which might lead to adipocyte dysfunction and metabolic diseases. The exact mechanism of such dysfunction requires further study to provide new insights into the pathogenesis of maternal obesity and the relevance of micronutrient supplementation for pregnant mothers.

Our study’s strength lies in comprehensively investigating human AT in two different depots complemented with in vitro cell line and primary adipocytes in a micronutrient deficient environment. However, there are some limitations. Although the study involved a large sample size, it is a cross-sectional study and from a single center. Hence, a longitudinal prospective cohort of women from early pregnancy with postpartum and offspring data is needed to evaluate whether mother’s gene expression on lipid and inflammation has influence in later-life and programs offspring cardiometabolic risk [8]. Socio-demographic and physiologic variables could influence the B12 levels and the expression of these genes in AT; therefore, inclusion of potential confounding variables and covariates in the regression analyses will predict whether maternal B12 independently associates with these metabolic risk factors by adjusting for likely confounders.

## Conclusions

In summary, our study evidenced that AT-specific B12 deficiency significantly dysregulated AT lipid metabolism and inflammation, thereby promoting dyslipidemia and worsening obesity-related complications that could underlie the development of IR and cardiometabolic risk. As B12 has a central role in methylation, further epigenetic studies are required to determine tissue-specific methylation changes related to lipid and inflammation pathways and metabolic phenotype. Overall, this study not only highlights the importance of B12 requirement in early pregnancy for improvement of metabolic status in mothers and offspring’s early life, but also warrants further study to reveal biological targets and mechanism for this high-risk sub-group of people for the later life treatment of metabolic disease in adulthood.

## Supplementary Information

Additional file 1: Tables 1–6: Show the correlation between biochemical variables and gene expression of enzymes involved in lipogenesis, fatty acid oxidation, and inflammatory cytokines in subcutaneous and omental adipose tissues.

## Data Availability

The data analysed in the current study are available from the corresponding author on reasonable request.

## Abbreviations

ACAA2: Acetyl-CoA acyltransferase
ACACA: Acetyl-CoA carboxylase alpha
ACADL: Acyl-CoA dehydrogenase long chain
AT: Adipose tissue
B12: Vitamin B12
BMI: Body mass index
CPT1β: Carnitine palmitoyltransferase-1
CPT2: Carnitine palmitoyltransferase-2
DGAT2: Diacylglycerol O-acyltransferase 2
ECHS1: Enoyl-CoA hydratase, short chain 1
ELOVL6: Elongation of very long fatty acid elongase 6
FA: Fatty acid
FAO: Fatty acid oxidation
FASN: Fatty acid synthase
FCCP: Carbonyl cyanide-p-tri-fluoro-methoxy-phenylhydrazone
GDM: Gestational diabetes mellitus
GPAT2: Glycerol-3-phosphate acyltransferase
HDL: High-density lipoprotein
hTERT: Human telomerase reverse transcriptase
IL- 1β: Interleukin-1 beta
IL- 6: Interleukin-6
IL- 18: Interleukin-18
KHB: Krebs-Henseleit Buffer
LIPIN1: Phosphatidate phosphatase
MCD: Malonyl-CoA decarboxylase
MCP- 1: Monocyte chemoattractant protein-1
OCR: Oxygen consumption rate
OmWAT: Omental white adipose tissue
SAM: S-adenosyl methionine
ScAT: Subcutaneous adipose tissue
SCD: Stearoyl-CoA desaturase
ScWAT: Subcutaneous white adipose tissue
T2D: Type 2 diabetes
TG: Triglyceride
TNFα: Tumor necrosis factor-α
UHCW: University Hospital Coventry and Warwickshire
WHO: World Health Organization

## References

[1] Langley-Evans SC, Pearce J, Ellis S. Overweight, obesity and excessive weight gain in pregnancy as risk factors for adverse pregnancy outcomes: a narrative review. J of Hum Nutri aDietcs. 2022;35(2):250–64. 10.1111/jhn.12999 PMC9311414 35239212

[2] Murray CJ, Aravkin AY, Zheng P, Abbafati C, Abbas KM, Abbasi-Kangevari M, Abd-Allah F, Abdelalim A, Abdollahi M, Abdollahpour I. Global burden of 87 risk factors in 204 countries and territories, 1990–2019: a systematic analysis for the global burden of disease study 2019. Lancet. 2020;396(10258):1223–49. 33069327 10.1016/S0140-6736(20)30752-2 PMC7566194

[3] Malnutrition in all its forms. https://www.emro.who.int/nutrition/double-burden-of-nutrition/index.html.

[4] Obesity profile: short statistical commentary May 2024. https://www.gov.uk/government/statistics/update-to-the-obesity-profile-on-fingertips/obesity-profile-short-statistical-commentary-may-2024.

[5] Kanasaki K, Kumagai A. The impact of micronutrient deficiency on pregnancy complications and development origin of health and disease. J Obst Gynae Res. 2021;47(6):1965–72. 10.1111/jog.14770 33783077

[6] McKay J, Ho S, Jane M, Pal S. Overweight & obese Australian adults and micronutrient deficiency. BMC nutri. 2020;6:1–13. 10.1186/s40795-020-00336-9 PMC7193396 32377370

[7] Niklewicz A, Smith AD, Smith A, Holzer A, Klein A, McCaddon A, Molloy AM, Wolffenbuttel BH, Nexo E, McNulty H. The importance of vitamin B12 for individuals choosing plant-based diets. Euro J Nutri. 2023;62(3):1551–9. 10.1007/s00394-022-03025-4 PMC10030528 36469110

[8] Saravanan P, Sukumar N, Adaikalakoteswari A, Goljan I, Venkataraman H, Gopinath A, Bagias C, Yajnik CS, Stallard N, Ghebremichael-Weldeselassie Y. Association of maternal vitamin B 12 and folate levels in early pregnancy with gestational diabetes: a prospective UK cohort study (PRiDE study). Diabetologia. 2021;64:2170–82. 34296321 10.1007/s00125-021-05510-7 PMC8423653

[9] Adaikalakoteswari A, Vatish M, Lawson A, Wood C, Sivakumar K, McTernan PG, Webster C, Anderson N, Yajnik CS, Tripathi G, et al. Low maternal vitamin B12 status is associated with lower cord blood HDL cholesterol in white Caucasians living in the UK. Nutrients. 2015;7(4):2401–14. 25849948 10.3390/nu7042401 PMC4425151

[10] Adaikalakoteswari A, Webster C, Goljan I, Saravanan P. Simultaneous detection of five one-carbon metabolites in plasma using stable isotope dilution liquid chromatography tandem mass spectrometry. J of Chroma B. 2016;1012:186–92. 10.1016/j.jchromb.2016.01.026 26851522

[11] Adaikalakoteswari A, Jayashri R, Sukumar N, Venkataraman H, Pradeepa R, Gokulakrishnan K, Anjana RM, McTernan PG, Tripathi G, Patel V. Vitamin B12 deficiency is associated with adverse lipid profile in Europeans and Indians with type 2 diabetes. Cardio Diabet. 2014;13(1):1–7. 10.1186/s12933-014-0129-4 PMC4189588 25283155

[12] Adaikalakoteswari A, Vatish M, Alam MT, Ott S, Kumar S, Saravanan P. Low vitamin B12 in pregnancy is associated with adipose-derived circulating miRs targeting PPAR γ and insulin resistance. J Clin Endo Metab. 2017;102(11):4200–9. 10.1210/jc.2017-01155 28938471

[13] Adaikalakoteswari A, Finer S, Voyias PD, McCarthy CM, Vatish M, Moore J, Smart-Halajko M, Bawazeer N, Al-Daghri NM, McTernan PG. Vitamin B12 insufficiency induces cholesterol biosynthesis by limiting s-adenosylmethionine and modulating the methylation of SREBF1 and LDLR genes. Clin Epigen. 2015;7(1):1–14. 10.1186/s13148-015-0046-8 PMC4356060 25763114

[14] Boachie J, Adaikalakoteswari A, Gázquez A, Zammit V, Larqué E, Saravanan P. Vitamin B12 induces hepatic fatty infiltration through altered fatty acid metabolism. Cel Physio Biochem. 2021;55(3):241–55. 10.33594/000000368 33961354

[15] Boachie J, Adaikalakoteswari A, Samavat J, Saravanan P. Low vitamin B12 and lipid metabolism: evidence from pre-clinical and clinical studies. Nutrients. 2020;12(7):1925. 32610503 10.3390/nu12071925 PMC7400011

[16] Al-Maskari MY, Waly MI, Ali A, Al-Shuaibi YS, Ouhtit A. Folate and vitamin B12 deficiency and hyperhomocysteinemia promote oxidative stress in adult type 2 diabetes. Nutrition. 2012;28(7–8):e23–6. 22595450 10.1016/j.nut.2012.01.005

[17] Ge Y, Yang C, Zadeh M, Sprague SM, Lin Y-D, Jain HS, Determann BF, Roth WH, Palavicini JP, Larochelle J. Functional regulation of microglia by vitamin B12 alleviates ischemic stroke-induced neuroinflammation in mice. Iscience. 2024;27:27(4). 10.1016/j.isci.2024.109480 PMC11075062 38715940

[18] Lee Y-J, Wang M-Y, Lin M-C, Lin P-T. Associations between vitamin B-12 status and oxidative stress and inflammation in diabetic vegetarians and omnivores. Nutrients. 2016;8(3):118. 26927168 10.3390/nu8030118 PMC4808848

[19] Harb Z, Deckert V, Bressenot AM, Christov C, Gueant-Rodriguez RM, Raso J, Alberto JM, de Barros JPP, Umoret R, Peyrin-Biroulet L. The deficit in folate and vitamin B12 triggers liver macrovesicular steatosis and inflammation in rats with dextran sodium sulfate-induced colitis. J Nutri Biochem. 2020;84:108415. 10.1016/j.jnutbio.2020.108415 32645655

[20] Ao M, Tsuji H, Shide K, Kosaka Y, Noda A, Inagaki N, Nakase H, Tanaka K. High prevalence of vitamin B-12 insufficiency in patients with Crohn’s disease. A P J Clin Nutri. 2017;26(6):1076–81. 10.6133/apjcn.022017.13 28917233

[21] Bressenot A, Pooya S, Bossenmeyer-Pourie C, Gauchotte G, Germain A, Chevaux J-B, Coste F, Vignaud J-M, Guéant J-L, Peyrin-Biroulet L. Methyl donor deficiency affects small-intestinal differentiation and barrier function in rats. Brit J Nutri. 2013;109(4):667–77. 10.1017/S0007114512001869 22794784

[22] Catapano AL, Pirillo A, Norata GD. Vascular inflammation and low-density lipoproteins: is cholesterol the link? A lesson from the clinical trials. Brit J Pharma. 2017;174(22):3973–85. 10.1111/bph.13805 PMC5659993 28369752

[23] Jung UJ, Choi M-S. Obesity and its metabolic complications: the role of adipokines and the relationship between obesity, inflammation, insulin resistance, dyslipidemia and nonalcoholic fatty liver disease. Int J Mol Sci. 2014;15(4):6184–223. 24733068 10.3390/ijms15046184 PMC4013623

[24] Jackisch L, Kumsaiyai W, Moore JD, Al-Daghri N, Kyrou I, Barber TM, Randeva H, Kumar S, Tripathi G, McTernan PG. Differential expression of Lp-PLA2 in obesity and type 2 diabetes and the influence of lipids. Diabetologia. 2018;61:1155–66. 29427237 10.1007/s00125-018-4558-6 PMC6449000

[25] Darimont C, Zbinden I, Avanti O, Leone-Vautravers P, Giusti V, Burckhardt P, Pfeifer A, Mace K. Reconstitution of telomerase activity combined with HPV-E7 expression allow human preadipocytes to preserve their differentiation capacity after immortalization. C D Differentiation. 2003;10(9):1025–31. 10.1038/sj.cdd.4401273 12934077

[26] Liu F, Kim J-k, Li Y, Liu X-q, Li J, Chen X. An extract of Lagerstroemia speciosa L. has insulin-like glucose uptake–stimulatory and adipocyte differentiation–inhibitory activities in 3T3-L1 cells. J Nutri. 2001;131(9):2242–7. 10.1093/jn/131.9.2242 11533261

[27] Gathercole LL, Bujalska IJ, Stewart PM, Tomlinson JW. Glucocorticoid modulation of insulin signaling in human subcutaneous adipose tissue. The J of Clin Endo Metab. 2007;92(11):4332–9. 10.1210/jc.2007-1399 PMC7611650 17711920

[28] Boachie J, Zammit V, Saravanan P, Adaikalakoteswari A. Metformin inefficiency to lower lipids in vitamin B12 deficient HepG2 cells is alleviated via adiponectin-AMPK axis. Nutrients. 2023;15(24):5046. 38140305 10.3390/nu15245046 PMC10745523

[29] Bradford MM. A rapid and sensitive method for the quantitation of microgram quantities of protein utilizing the principle of protein-dye binding. Ana Biochem. 1976;72(1–2):248–54. 10.1016/0003-2697(76)90527-3 942051

[30] Qin S, Wang Y, Li L, Liu J, Xiao C, Duan D, et al. Early-life vitamin B12 orchestrates lipid peroxidation to ensure reproductive success via SBP-1/SREBP1 in Caenorhabditis elegans. Cell Rep. 2022;40(12):111381. 10.1016/j.celrep.2022.111381. 10.1016/j.celrep.2022.111381 36130518

[31] Marcelin G, Chua S Jr. Contributions of adipocyte lipid metabolism to body fat content and implications for the treatment of obesity. Curr Opin in Pharma. 2010;10(5):588–93. 10.1016/j.coph.2010.05.008 PMC2945394 20860920

[32] Houmard JA. Intramuscular lipid oxidation and obesity. A J of Physio-Reg, Integ and Comp Physio. 2008;294(4):R1111–6. 10.1152/ajpregu.00396.2007 PMC2367219 18256136

[33] Annesley SJ, Fisher PR. Mitochondria in health and disease. MDPI. 2019;8:680. 10.3390/cells8070680 PMC6678092 31284394

[34] Pooya S, Blaise S, Garcia MM, Giudicelli J, Alberto J-M, Guéant-Rodriguez R-M, Jeannesson E, Gueguen N, Bressenot A, Nicolas B. Methyl donor deficiency impairs fatty acid oxidation through PGC-1α hypomethylation and decreased ER-α, ERR-α, and HNF-4α in the rat liver. J of hepato. 2012;57(2):344–51. 10.1016/j.jhep.2012.03.028 22521344

[35] Dahlhoff C, Worsch S, Sailer M, Hummel BA, Fiamoncini J, Uebel K, Obeid R, Scherling C, Geisel J, Bader BL. Methyl-donor supplementation in obese mice prevents the progression of NAFLD, activates AMPK and decreases acyl-carnitine levels. Mol Metabol. 2014;3(5):565–80. 10.1016/j.molmet.2014.04.010 PMC4099513 25061561

[36] Domínguez-López I, Kovatcheva M, Casas R, Toledo E, Fitó M, Ros E, Estruch R, Serrano M, Lamuela-Raventós RM. Higher circulating vitamin B12 is associated with lower levels of inflammatory markers in individuals at high cardiovascular risk and in naturally aged mice. J of the Sci of Food and Agri. 2024;104(2):875–82. 10.1002/jsfa.12976 37690097

[37] Al-Daghri NM, Rahman S, Sabico S, Yakout S, Wani K, Al-Attas OS, Saravanan P, Tripathi G, McTernan PG, Alokail MS. Association of vitamin B12 with pro-inflammatory cytokines and biochemical markers related to cardiometabolic risk in Saudi subjects. Nutrients. 2016;8(9):460. 27608037 10.3390/nu8090460 PMC5037505

[38] Vaccaro JA, Qasem A, Naser SA. Folate and vitamin B12 deficiency exacerbate inflammation during Mycobacterium avium paratuberculosis (MAP) infection. Nutrients. 2023;15(2):261. 36678131 10.3390/nu15020261 PMC9865721

[39] Tripathi M, Singh BK, Zhou J, Tikno K, Widjaja A, Sandireddy R, Arul K, Ghani SABA, Bee GGB, Wong KA. Vitamin B12 and folate decrease inflammation and fibrosis in NASH by preventing syntaxin 17 homocysteinylation. J of Hepato. 2022;77(5):1246–55. 10.1016/j.jhep.2022.06.033 35820507

[40] Guest J, Bilgin A, Hokin B, Mori TA, Croft KD, Grant R. Novel relationships between B12, folate and markers of inflammation, oxidative stress and NAD (H) levels, systemically and in the CNS of a healthy human cohort. Nutri Neuro. 2015;18(8):355–64. 10.1179/1476830515Y.0000000041 26263423

