# Supplementary material for: Maternal B12 deficiency during pregnancy dysregulates fatty acid metabolism and induces inflammation in human adipose tissue
Source: BMC Med. 2025 Apr 23;23:232. doi: 10.1186/s12916-025-04056-4 (PMC12016209; doi:10.1186/s12916-025-04056-4)
Supplement: Supplementary file 1 — Additional file 1: Tables 1–6: Show the correlation between biochemical variables and gene expression of enzymes involved in lipogenesis, fatty acid oxidation, and inflammatory cytokines in subcutaneous and omental adipose tissues. [file 12916_2025_4056_MOESM1_ESM.docx]

| **Relative Subcutaneous mRNA**  **expression** | **Serum biochemical metabolites** | | | | | |
| --- | --- | --- | --- | --- | --- | --- |
|  | **B12**  **(pM)** | **Glucose**  **(mM)** | **Cholesterol (mM)** | **HDL**  **(mM)** | **LDL**  **(mM)** | **Triglycerides (mM)** |
| **ACACA** | **-0.234*** | 0.115 | **0.201*** | 0.002 | **0.221*** | **0.211*** |
| **FASN** | **-0.205*** | -0.040 | 0.092 | 0.097 | 0.107 | 0.083 |
| **ELOVL6** | 0.028 | 0.060 | 0.011 | 0.136 | 0.005 | -0.057 |
| **SCD** | 0.009 | 0.048 | -0.013 | 0.066 | 0.021 | -0.092 |
| **GPAT2** | -0.100 | **0.222*** | -0.053 | 0.003 | -0.073 | -0.042 |
| **LIPIN1** | -0.149 | -0.025 | -0.086 | -0.064 | -0.051 | -0.163 |
| **DGAT2** | 0.132 | 0.102 | 0.066 | -0.000 | 0.075 | -0.097 |

**Additional File 1: Table 1: mRNA expression of genes involved in subcutaneous lipid synthesis correlates with serum biochemical metabolites**

| **Relative Omental mRNA**  **expression** | **Serum biochemical metabolites** | | | | | |
| --- | --- | --- | --- | --- | --- | --- |
|  | **B12**  **(pM)** | **Glucose (mM)** | **Chol. (mM)** | **HDL**  **(mM)** | **LDL**  **(mM)** | **Triglycerides (mM)** |
| **ACACA** | **-0.235*** | -0.084 | 0.047 | **-0.209*** | 0.085 | 0.024 |
| **FASN** | **-0.261**** | -0.040 | -0.032 | -0.090 | -0.046 | 0.220 |
| **ELOVL6** | -0.054 | -0.118 | **0.247**** | -0.010 | **0.298**** | **0.210*** |
| **SCD** | **-0.203*** | -0.113 | 0.023 | -0.102 | 0.105 | **0.220*** |
| **GPAT2** | **-0.192*** | -0.028 | -0.051 | -0.014 | -0.109 | -0.001 |
| **LIPIN1** | **-0.201*** | -0.038 | -0.103 | **-0.217*** | -0.071 | 0.009 |
| **DGAT2** | **-0.262**** | 0.004 | 0.018 | 0.044 | 0.035 | **0.194*** |

**Additional File 1: Table 2: mRNA expression of genes involved in omental lipid synthesis correlates with serum biochemical metabolites**

| **Relative**  **Subcutaneous mRNA expression** | **Serum biochemical values** | | | |
| --- | --- | --- | --- | --- |
|  | **B12**  **(pM )** | **Glucose (mM)** | **Triglycerides (mM)** | **HDL**  **(mM)** |
| **MCD** | **0.203*** | 0.041 | 0.022 | 0.129 |
| **CPT1β** | **0.212*** | 0.010 | 0.086 | -0.114 |
| **CPT2** | **0.325***** | -0.062 | -0.067 | **0.231*** |
| **ACADL** | **0.213*** | 0.014 | 0.048 | 0.114 |
| **ECHS1** | **0.222*** | 0.052 | -0.009 | **0.192*** |
| **ACAA2** | **0.200*** | 0.014 | 0.048 | 0.114 |

**Additional File 1: Table 3**: **Subcutaneous fatty acid β-oxidation gene expression correlates with serum biochemical metabolites**:

| **Relative Omental mRNA expression** | **Serum biochemical values** | | | |
| --- | --- | --- | --- | --- |
|  | **B12**  **(pM)** | **Glucose**  **(mM)** | **Triglycerides**  **(mM)** | **HDL**  **(mM)** |
| **MCD** | **0.199*** | -0.050 | 0.113 | -0.072 |
| **CPT1β** | **0.209*** | 0.155 | 0.047 | 0.019 |
| **CPT2** | **0.195*** | **-0.202*** | **-0.217*** | 0.106 |
| **ACADL** | 0.004 | 0.074 | 0.079 | 0.088 |
| **ECHS1** | 0.095 | 0.007 | -0.066 | 0.113 |
| **ACAA2** | **0.289**** | 0.023 | **-0.215*** | -0.025 |

**Additional File 1: Table 4: Omental fatty acid β-oxidation gene expression correlates with serum biochemical metabolites**:

| **Relative Sub. mRNA**  **expressi on** | **Serum biochemical values** | | | | | | |
| --- | --- | --- | --- | --- | --- | --- | --- |
|  | **Glucose (mM)** | **Cholesterol**  **(mM)** | **HDL**  **(mM)** | **LDL**  **(mM)** | **Triglyce rides (mM)** | **MCP-1**  **(pg/ml)** | **IL-8**  **(pg/ml)** |
| **IL-1β** | 0.080 | 0.136 | 0.056 | **0.263**** | -0.063 | **0.325**** | **0.234*** |
| **IL-6** | 0.097 | 0.025 | -0.029 | 0.026 | 0.026 | -0.000 | 0.132 |
| **IL-8** | **0.206*** | -0.109 | **-0.203*** | 0.012 | **0.201*** | 0.138 | 0.121 |
| **IL-18** | 0.120 | -0.005 | -0.022 | 0.028 | -0.030 | 0.009 | 0.122 |
| **MCP-1** | -0.002 | -0.063 | **-0.255**** | 0.011 | 0.134 | -0.015 | 0.054 |
| **TGF-β** | -0.087 | -0.026 | 0.082 | -0.060 | -0.073 | 0.120 | 0.135 |
| **TNF-α** | -0.031 | 0.156 | 0.110 | 0.138 | 0.030 | 0.006 | 0.0061 |

**Additional File 1: Table 5: Subcutaneous inflammatory gene expression correlates with serum biochemical metabolites**

| **Relative Omental mRNA**  **expression** | **Serum biochemical values** | | | | | | |
| --- | --- | --- | --- | --- | --- | --- | --- |
|  |  |  |  |  |  |  |  |
|  | **Glucose (mM)** | **Cholesterol**  **(mM)** | **HDL**  **(mM)** | **LDL**  **(mM)** | **Triglycerides (mM)** | **MCP-1**  **(pg/ml)** | **IL-8**  **(pg/ml)** |
|  |  |  |  |  |  |  |  |
|  |  |  |  |  |  |  |  |
| **IL-1β** | -0.060 | **0.196*** | 0.027 | **0.212*** | -0.040 | **-0.250*** | -0.013 |
| **IL-6** | -0.083 | 0.077 | 0.070 | **0.203*** | -0.044 | -0.083 | **-0.257*** |
| **IL-8** | **-0.206*** | -0.109 | **-0.203*** | 0.012 | 0.020 | 0.138 | 0.121 |
| **IL-18** | 0.006 | 0.029 | **-**  **0.333***** | 0.122 | **0.361**** | 0.160 | **0.232*** |
| **MCP-1** | 0.113 | -0.008 | -0.084 | 0.090 | -0.033 | 0.145 | 0.030 |
| **TGF-β** | 0.049 | **-0.232*** | -0.091 | **-0.215*** | -0.061 | 0.140 | 0.140 |
| **TNF-α** | **0.203*** | -0.037 | **0.198*** | -0.070 | -0.029 | 0.077 | 0.069 |

**Additional File 1: Table 6: Omental inflammatory gene expression correlates with serum biochemical metabolites**
